# Return to work after total hip and knee arthroplasty: results from a clinical study

**DOI:** 10.1007/s00296-015-3311-4

**Published:** 2015-06-29

**Authors:** C. Tilbury, C. S. Leichtenberg, R. L. Tordoir, M. J. Holtslag, S. H. M. Verdegaal, H. M. Kroon, R. G. H. H. Nelissen, T. P. M. Vliet Vlieland

**Affiliations:** Department of Orthopaedics J11, Leiden University Medical Center, P.O. Box 9600, 2300 RC Leiden, The Netherlands; Department of Orthopaedics, Alrijne Hospital, Leiderdorp, The Netherlands; Department of Radiology, Leiden University Medical Center, Leiden, The Netherlands

**Keywords:** Arthroplasty, Employment, Work, Total hip, Total knee, Sick leave

## Abstract

The aim of this study was to measure return to work and duration until return to work in patients undergoing total hip or knee arthroplasty (THA or TKA). This prospective study included patients under 65 years of age, undergoing THA or TKA, who provided information on their work status preoperatively (paid work yes/no and working hours) and 1 year thereafter (paid work yes/no, working hours and time until return to work). Seventy-one THA and 64 TKA patients had a paid job preoperatively. The employment rates 1 year postoperatively were 64/71 (90 %) after THA and 53/64 (83 %) after TKA. Of those who returned to work, 9/64 (14 %) of THA patients and 10/53 (19 %) of TKA patients worked less hours than preoperatively [mean decrease of 16 (SD 11.5) and 14 (SD 13.0) hours, respectively]. The mean time to return to work was 12.5 (SD 7.6) and 12.9 (SD 8.0) weeks in THA and TKA, respectively. The majority of working patients who underwent THA or TKA returned to work, after approximately 12 weeks. A considerable proportion of the patients returning to work worked less hours than preoperatively. More research into patients who do not return or decrease their working hours is needed.

## Introduction

Total hip arthroplasty (THA) and total knee arthroplasty (TKA) are very effective procedures to improve pain and functioning in patients with hip and knee osteoarthritis [1, 2]. The numbers of patients undergoing THA and TKA surgery are substantial, the rate per 100.000 persons varying between 70 and 112 in northern European countries and the USA [3–5]. Although the rates reported in the literature vary, there are many studies showing that a considerable proportion of these patients (15–45 % [6, 7, 9]) is of working age and/or <65 years at the time of surgery.

With respect to return to work after THA or TKA, the number of studies is limited. A recent systematic review by Tilbury et al. [6], including studies from 1986 to 2013, found that the majority of patients who are employed before THA and TKA returned to work postoperatively. Only few of the studies included in this review reported the mean time to return to work, with the reported durations ranging from 1.1 to 10.5 weeks after THA [1, 7–11] and from 8.0 to 12.0 weeks after TKA [2, 11, 12]. As the study designs as well as the assessment methods varied largely among the studies, firm conclusions regarding the speed of return to work cannot be drawn [6].

After this systematic review was completed, a study by Sankar et al. [13] was published, evaluating the return to work among 360 THA and TKA patients who were working preoperatively or on a short-term disability pension. It was found that 87 % of THA and 85 % of TKA patients had returned to work 1 year after surgery. This study did not report the mean time to return to work. Kievit et al. [14] examined the impact of TKA on patients’ reintegration into the workplace, showing that 117 of 173 working patients (68 %) had returned to work 3.8 (1.3 SD) years after surgery. Lombardi et al. [15] found in a group of 494 patients who were employed before TKA that 98 % returned to work after on average 8.9 weeks (SD 9.1).

Concerning beneficial and limiting factors affecting return to work after surgery, Kuijer et al. [16] conducted a systematic review including studies published between 1998 and 2008. All of the three studies included in that review concerned THA, with the results suggesting that using a two-incision approach has a beneficial effect on return to work, whereas the provision of movement restrictions had a negative effect, and patient discharge guidelines had no effect on the time to return to work. In the review by our own group, factors related to work status after THA and TKA included sociodemographic, health and job characteristics [6].

For the appropriate timing of interventions aiming to foster return to work, insight into the course of work disability after THA and TKA is needed. Given the lack of knowledge on the time to return to work after joint arthroplasties, the aim of the present study was to describe the work status and the duration until return to work after THA and TKA. Moreover, characteristics of patients who did and did not return to work were compared.

## Methods

### Study design

This study on return to work was part of a prospective cohort study on the outcomes of THA and TKA performed at the Department of Orthopedics of the Alrijne Hospital (former Rijnland Hospital), the Netherlands, from October 2010 to September 2013 (inclusion of patients was done until September 2012). The study protocol was reviewed and approved by the local hospital Review Board of the Alrijne Hospital, Leiderdorp in the Netherlands (registration number 10/07), which is attached to the Medical Research Ethics Committee of the Leiden University Medical Center, Leiden, the Netherlands. Of all patients, written informed consent to participate in the study was obtained. Funding for the present study on return to work was received from the Anna Fonds, NOREF (Dutch Association Orthopaedic Research and Education Foundation) and the Dutch Arthritis Association LRR.

### Patients and recruitment

The prospective cohort study aimed to include all consecutive patients undergoing a primary THA or TKA because of osteoarthritis, aged 18 years or older, able to read and understand Dutch and being mentally and physically able to complete questionnaires. Excluded were patients with revision of a THA or TKA, undergoing a hemi-arthroplasty and undergoing a THA or TKA because of tumor or rheumatoid arthritis.

One day preoperatively, before being admitted to the hospital, the treating orthopedic surgeon provided oral and written information on the study to all eligible patients.

For the present study on return to work, only the data from patients under the age of 65 years (the retirement age in the Netherlands at the time the study was conducted) were used.

## Assessments

The preoperative questionnaires were administered by the treating physician, and the postoperative questionnaire was sent by regular mail. A telephone interview was scheduled if the answers regarding work status in either the preoperative or follow-up questionnaires were incomplete. These telephone interviews were conducted by one of the researchers (CSL). Sociodemographic and general patient characteristics were only gathered preoperatively.

### Sociodemographic and general patient characteristics

Sociodemographic characteristics were recorded preoperatively and included: age (years), sex, length (cm) and weight (kg) to calculate the body mass index, level of education (low: primary school, lower vocational education; medium: lower general secondary school, intermediate vocational education; or high: higher general secondary school, higher vocational education, university) and marital status (living alone; yes/no).

### Work status

At the preoperative assessment, all patients were asked to indicate whether they had a paid job (yes/no). If not, they were asked to indicate whether they were pensioner, housewife/houseman or unemployed.

If they were working, they were requested to provide information on the following aspects of their working situation: (a) amount of hours currently working per week; (b) being self-employed or wage earner; (c) current complete or partial sick leave or complete or partial sick leave over the past 12 months, with sick leave defined as absenteeism related to the hip or knee complaints and reported to the employer; if yes, duration of 4 weeks or more (yes/no); (d) presence of work adaptions yes/no; if yes: change in tasks, performing fewer tasks, changes in working hours or other work-related adaptions or devices (all these questions could be answered with yes or no); (e) receipt of partial disability benefits related to hip or knee complaints (yes/no).

In the follow-up assessment, the same questions were used, with in addition: (f) working currently (yes/no); (g) duration until return to work for the first time (weeks); (h) number of hours working per week when starting to work for the first time; and (i) numbers of hours working per week after 1 year. If the follow-up questionnaire was returned incomplete, patients were contacted by telephone to provide the required information.

### Health related quality of life

The Short Form 36 Health Survey questionnaire (SF-36) is composed of 36 questions and standardized response choices, organized into eight multi-item scales: physical functioning (PF), role limitations due to physical health problems (RP), bodily pain (BP), general health perceptions (GH), vitality (VT), social functioning (SF), role limitations due to emotional problems (RE) and general mental health (MH). From these eight subscales, the SF-36 mental and physical component scales (MCS and PCS) were computed. For that purpose, the method of norm-based scoring was used [17]. In norm-based scoring, each scale is scored to have the same average (mean: 50) and the same standard deviation (SD: 10), meaning each point equals one-tenth of a standard deviation. In this study, scores of a Dutch general population [18] were used to standardize the scores according to the method of norm-based scoring. Lower scores represent worse health status.

The Euroqol-5 Dimension (EQ-5D) is an instrument designed to derive from five dimensions of health (mobility, self-care, usual activities, pain and mood), a single cardinal index for the quality weighting of QALYs. The EQ-5D uses valuations derived with the time trade-off method from a large general population survey to score the five-dimension health profile self-reported. The second part Euroqol visual analogue scale (EQ-VAS) consists of a 20-cm vertical visual analogue scale (VAS) ranging from 100 (best imaginable health state) to 0 (worst imaginable health state). The EQ-VAS gives a self-assessed measure of overall health state [19].

### Functional outcome measurement

Hip and knee functions were assessed by means of the following outcome measures:

- The Hip disability and Osteoarthritis Outcome Score (HOOS), consisting of 40 items divided over five dimensions: pain (P) (10 items), symptoms (S) including stiffness and range of motion (5 items), activity limitations—daily living (A) (17 items), sport and recreation function (SP) (4 items) and hip-related quality of life (Q) (4 items) [20] The Knee injury and Osteoarthritis Outcome Score (KOOS) comprises 42 items and uses the same five subscales as the HOOS [21]. For the present study, validated Dutch versions of the HOOS and KOOS were used [22].
- The Oxford Hip Score (OHS) and the Oxford Knee Score (OKS), which are short, twelve-item questionnaires developed for completion by patients undergoing THA and TKA [23, 24]. We used validated Dutch translations for the present study [25, 26].

### Preoperative radiological severity

Preoperative supine radiographs of hips (anterior–posterior) and weight-bearing radiographs of the knees (posterior–anterior) were collected from the patients’ medical records. These radiographs were routinely made in the participating centers for preoperative templating purposes. All radiographs were assessed by an experienced musculoskeletal radiologist (HMK), who was blinded for the operated side and patient characteristics. The Kellgren and Lawrence (KL) grading system was used to classify the severity of OA (grade 0: no OA; grade 1: doubtful OA; grade 2: minimal OA; grade 3: moderate OA and grade 4: severe OA) [27]. Ten percentage of the radiographs were scored twice: correlation between both readings was used to establish intra-reader reliability [intra-class correlation hip radiographs: 99 % (95 % CI 85–93 %); intra-class correlation knee radiographs: 95 % (95 % CI 92–98 %)]. The second reading was used for further statistical analyses. The KL grade in our study was classified as KL 0–1 (no OA), KL 2 (mild OA) and KL 3–4 (severe OA).

### Statistical analyses

Descriptive statistics were used to present the characteristics of patients and their working status preoperatively and at follow-up. Comparisons of the baseline characteristics between working patients and patients who were not working at the preoperative assessment were made by means of the Mann–Whitney *U* test or Chi-square test. For all clinical outcome measures, change scores between the preoperative assessment and 1-year follow-up were computed with the 95 % confidence interval. Comparisons of working hours before and after surgery within the group of working patients were made by means of the Wilcoxon signed-rank test. Sociodemographic and job characteristics and patient-reported outcomes (SF-36, EQ-5D, EQ5D-VAS and HOOS/KOOS) were compared between patients who were working preoperatively and did return to work and patients who did not, by means of the Mann–Whitney *U* test or Chi-square test, where appropriate. All data were analyzed using the SPSS statistical package (version 20.0, SPSS, Chicago, Illinois). The level of statistical significance was set at *p* ≤ 0.05 for all analyses.

## Results

**Fig. 1:**
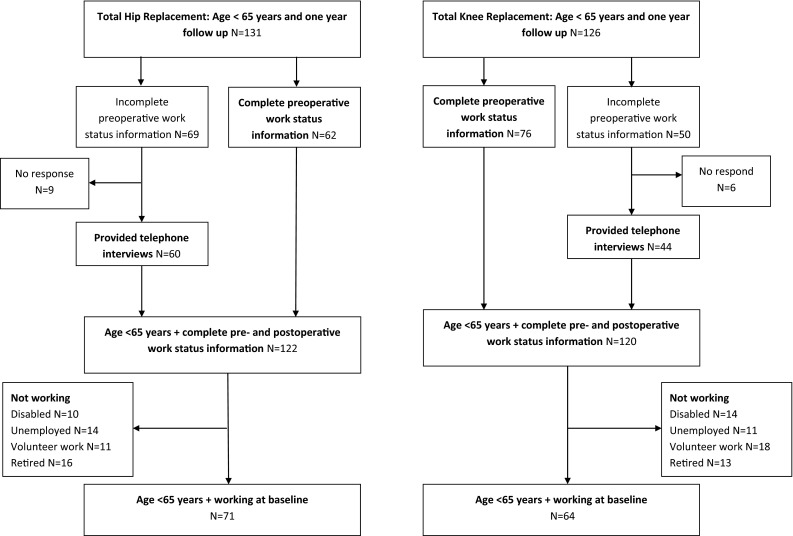
Flow of patients participating in a cohort study on outcomes of total hip and total knee arthroplasty

In the larger study, 428 THA and 417 TKA patients were included of whom 343 THA (80 %) and 322 TKA (77 %) completed the postoperative questionnaire. Of these, 131 THA patients (38 %) and 126 of TKA patients (39 %) were under 65 years. Figure 1 describes the flow of the patients included in the present analysis. Information regarding preoperative work status of 69 THA patients (53 %) and 50 TKA patients (40 %) was incomplete or inconclusive; these 119 patients were approached for additional telephone interviews. Fifteen patients (13 %) could not be reached and were therefore excluded for the present analyses. This resulted in 122 THA patients (93 %) and 120 TKA patients (95 %) who were under 65 years and provided complete information on their work status preoperatively.

### Preoperative work status and characteristics of working and non-working patients

The preoperative work status of patients under 65 years is described in Table 1. The mean age of the 122 THA patients was 57.7 years (6.3 SD) and of the 120 TKA patients 57.4 years (5.8 SD). There were 70 females (57 %) in the THA group and 79 (66 %) in the TKA group. Preoperatively, 71 of 122 THA patients (58 %) and 64 of 120 TKA patients (53 %) were working; 14 THA patients (11 %) and 11 TKA patients (8 %) were unemployed and/or looking for a job; 10 THA patients (8 %) and 14 TKA patients (12 %) were disabled, of those 10 THA patients (8 %) and 8 TKA patients (13 %) received a full disability pension; 2 THA patients (3 %) and 5 TKA patients (8 %) received disability benefits because of hip or knee impairments; 11 THA patients (9 %) and 18 TKA patients (15 %) were doing household and/or volunteer work; and 16 THA patients (13 %) and 13 TKA patients (8 %) were retired (see Table 1).

**Table 1:** Characteristics of patients <65 years of age undergoing THA and TKA participating in a prospective cohort study

Variable	THA patients employed (*N* = 71)	THA patients not working (unemployed/looking for a job, disability pension, retired, volunteer work) (*N* = 51)	^#^ *P* value	TKA patients employed (*N* = 64)	TKA patients not working (unemployed/looking for a job, disability pension, retired, volunteer work) (*N* = 56)	^#^ *P* value
Sex, female, *n* (%)	34 (48 %)	36 (71 %)	0.012*	34 (53 %)	45 (80 %)	0.002*
Age [years, mean (SD)]	56.0 (6.6)	60.0 (5.0)	0.000*	56.2 (5.8)	58.8 (5.7)	0.002*
Age, categories, *N* (%)						
18–45	8 (11 %)	2 (4 %)		4 (6 %)	4 (7 %)	
46–55	17 (24 %)	5 (10 %)		17 (27 %)	6 (11 %)	
56–65	46 (65 %)	44 (86 %)		42 (67 %)	46 (82 %)	
Body mass index, mean (SD)	27.8 (6.0)	26.8 (4.2)	0.739	29.9 (4.5)	30.5 (5.1)	0.690
BMI, categories, *N* (%)	20 (30 %)	13 (29 %)		6 (10 %)	5 (10 %)	
Normal 18.5–24.9	32 (48 %)	19 (42 %)		22 (37 %)	22 (43 %)	
Overweight 25–29.9	10 (15 %)	12 (27 %)		23 (39 %)	16 (31 %)	
Obese 30+	5 (7 %)	1 (2 %)		8 (14 %)	8 (16 %)	
Education level, *n* (%)						
Low	28 (40 %)	30 (60 %)	0.098	33 (52 %)	42 (75 %)	0.010*
Medium	19 (27 %)	7 (14 %)		12 (19 %)	9 (16 %)	
High	23 (33 %)	14 (28 %)		19 (30 %)	5 (9 %)	
Radiographic severity, *n* (%)						
KL grade 0–1	4 (6 %)	3 (8 %)	0.974	5 (10 %)	4 (8 %)	0.132
KL grade 2	13 (21 %)	8 (20 %)		5 (10 %)	12 (26 %)	
KL grade 3–4	46 (73 %)	29 (73 %)		39 (80 %)	31 (66 %)	
Living status						
Living Independently, yes, *n* (%)	71 (100 %)			63 (100 %)	55 (100 %)	
HOOS or KOOS, mean (SD)						
ADL	42 (17.5)	45 (19.0)	0.437	43.8 (16.1)	46.3 (16.1)	0.481
Pain	39 (20.3)	43 (17.1)	0.174	35.4 (14.8)	36.0 (12.9)	0.917
Quality of life	33 (9.1)	35 (8.8)	0.261	30.3 (9.2)	33.9 (8.7)	0.055
Sport and recreation	16 (17.6)	20 (15.8)	0.087	8.3 (10.6)	9.9 (12.4)	0.529
Symptoms	32 (18.9)	36 (17.7)	0.172	42.9 (14.5)	41.6 (14.2)	0.441
EQ-5D score, mean (SD)	0.5 (0.3)	0.6 (0.3)	0.858	0.5 (0.3)	0.6 (0.3)	0.097
EQ-VAS, mean (SD)	62 (18.7)	63 (20.2)	0.600	64.7 (18.3)	72.1 (18.1)	0.030*
Oxford Knee/Hip Score, mean (SD)	23 (7.0)	26 (6.9)	0.048*	24.0 (6.9)	23.3 (5.8)	0.655
SF-36 MCS, mean (SD)	51 (10.5)	49 (11.8)	0.863	53.2 (9.8)	50.5 (13.0)	0.084
SF-36 PCS, mean (SD)	40 (6.8)	41 (8.4)	0.571	38.5 (7.4)	40.8 (6.3)	0.381

*HOOS* Hip Disability and Osteoarthritis Outcome Score, *KOOS* Knee Injury and Osteoarthritis Outcome Score, *ADL* Activities limitations—Daily Living, *EQ-5D* EuroQol-5 Dimension, *EQ-VAS* EuroQol visual analogue scale, *SF-36 MCS* Short Form 36 Mental Component Summary Scale, *SF-36 PCS* Short Form 36 Physical Component Summary Scale

^#^Comparison of working and non-working patients at preoperative assessment by means of Mann–Whitney *U* or Chi-square tests where appropriate. * Significance level <0.05

Table 1 also describes the clinical characteristics of working and non-working patients undergoing THA and TKA. In the THA group, working patients were significantly more often male, though in the TKA group female. In both groups, the working patients were significantly younger than the non-working patients, whereas in the TKA group the working patients were higher educated and in the THA group the Oxford Hip Score was significantly lower in working patients. No other statistically significant differences regarding the characteristics of working and non-working patients were seen.

### Characteristics of preoperative work situation in working patients

Table 2 describes the characteristics of preoperative work situation in the working 71 THA and 64 TKA patients.

**Table 2:** Characteristics of preoperative work situation in working patients undergoing total hip or knee arthroplasty (THA or TKA)

	THA (*N* = 71)	TKA (*N* = 64)
Self-employed, *N* (%)	12 (17 %)	9 (16 %)
Hours working per week preoperatively, mean (SD)	32 (12.7)	31 (12.7)
Absence from work in connection with the hip/knee complaints in last year, *N* (%)		
Not at all	43 (60 %)	36 (56 %)
Less than 4 weeks	7 (10 %)	6 (9 %)
More than 4 weeks	12 (17 %)	12 (19 %)
Unknown	9 (13 %)	10 (16 %)
Adaptions at work, *N* (%)		
None	37 (77 %)	33 (52 %)
Different tasks	3 (6 %)	7 (11 %)
Less tasks	6 (9 %)	3 (5 %)
Change of working hours	1 (2 %)	4 (6 %)
Work-related adaptions or devices	1 (2 %)	2 (3 %)
Unknown	–	15 (23 %)
Receiving workers compensation, *N* (%)		
None	57 (80 %)	51 (80 %)
Yes, in connection with the hip/knee complaints	2 (3 %)	4 (6 %)
Yes, in connection with other health complaints	3 (4 %)	3 (5 %)
Unknown	9 (13 %)	6 (9 %)

Both in THA and in TKA patients, most preoperatively working patients were wage earners. The mean number of working hours preoperatively was 32 h in THA patients (SD 12.7) and 31 h in TKA patients (SD 12.7). In the 63 and 55 THA and TKA patients in whom both the preoperative and postoperative number of working hours were known, paired comparisons showed a statistically significant decrease (*p* = 0.04 and *p* = 0.02). Of the working patients, 43 THA patients (61 %) and 36 TKA patients (56 %) had not been absent from work in the past year related to their hip or knee complaints. Twelve THA patients (17 %) and 12 TKA patients (19 %) had been absent from work for more than 4 weeks. Fifty-seven working THA patients (80 %) and 51 working TKA patients (80 %) indicated that their work had not been adjusted because of the hip or knee complaints preoperatively. The other patients either did different tasks, performed less tasks, worked different hours or received work-related adaptions or devices. Of the preoperatively working patients, slightly more than half had been in contact with an occupational physician about return to work either preoperatively or postoperatively.

### Return to work and clinical outcomes

Table 3 describes the work status and changes in clinical outcome measures 1 year after THA or TKA in patients who were working preoperatively. Two and five patients who were working preoperatively were retired 1 year after surgery in the THA and TKA groups, respectively. For the 64 and 56 patients who were working both preoperatively and 1 year thereafter, the mean time to return to work was 12.5 weeks (SD 7.6; median 12; minimum 1; maximum 40 weeks) and 12.9 weeks (SD 8.0; median 12; minimum 1; maximum 36 weeks) in the THA and TKA groups, respectively. Of the 64 and 53 patients returning to work of whom the number of hours working per week 1 year postoperatively was known, 9 (14 %) and 10 (19 %) patients worked less hours than preoperatively in the THA and TKA groups (mean decrease of 16 (SD 11.5; minimum 5; maximum 35) and 14 (SD 12.5; minimum 2; maximum 38) hours, respectively). Comparison of working hours before and after surgery shows significant differences in both THA (*p* = 0.044) and TKA (*p* = 0.018).

**Table 3:** Return to work 1 year postoperatively and change scores with the 95 % confidence interval (CI) of clinical outcomes in working patients undergoing total hip or knee arthroplasty (THA or TKA)

	THA (*N* = 71)	TKA (*n* = 64)
Working situation, *N* (%)		
Returned to work	64 (90 %)	56 (89 %)
Sick leave	2 (3 %)	3 (5 %)
Retired	2 (3 %)	5 (8 %)
Unknown	3 (4 %)	0
Amount of weeks between operation and return to work		
Mean (SD)	12.5 (7.6)	12.9 (8.0)
Median (minimum–maximum)	12 (1–40)	12 (1–36)
Hours working per week postoperatively, mean (SD)	30 (12.0)	26 (12.9)
Been in contact with the occupational physician about return to work, *N* (%)		
Yes	39 (55 %)	39 (61 %)
No	26 (37 %)	13 (20 %)
Unknown	6 (8 %)	12 (19 %)
HOOS or KOOS change scores, mean (95 % CI)		
ADL	49 (44–54)*	36 (31–43)*
Pain	53 (47–58)*	43 (37–49)*
Quality of life	19 (15–24)*	16 (10–21)*
Sport	51 (44–59)*	34 (26–42)*
Symptoms	51 (45–58)*	7 (3–11)*
Oxford Knee/Hip change score, mean (95 % CI)	20 (18–22)*	15 (13–18)*
EQ-5D change score, mean (95 % CI)	0.3 (0.2–0.4)*	0.3 (0.2–0.3)*
EQ5D-VAS scale change score, mean (95 % CI)	20 (14–26)*	13 (7–18)*
SF-36 MCS change score, mean (SD; min–max)	1.3 (−1.3–3.7)	−0.9 (−4–2)
SF-36 PCS change score, mean (SD; min–max)	14.9 (13–17)*	12 (9–15)*

*HOOS* Hip disability and Osteoarthritis Outcome Score, *KOOS* Knee injury and Osteoarthritis Score, *ADL* Activity limitations—Daily Living, *SF-36* Short Form 36

* Comparison of clinical outcomes before and after surgery was made by means of paired *t* test

All clinical outcome measures, except for the SF-36 MCS, showed a statistically significant change over time, both in the THA and in the TKA groups.

One year after surgery, there were seven patients (three and four in the THA and TKA groups, respectively) who were working but had not been gainfully employed preoperatively. The preoperative employment status of these patients included: receiving a disability pension (*n* = 1), unemployed (*n* = 1) and doing volunteer work (*n* = 1) in the THA group and receiving a disability pension (*n* = 1), being retired (*n* = 1), unemployed (*n* = 1) and doing volunteer work (*n* = 1) in the TKA group.

### Characteristics of patients returning and not returning to work

A comparison of the sociodemographic (gender, age, BMI, education level, living status), job characteristics and patient-reported outcomes (preoperative SF-36, EQ-5D, EQ5D-VAS and HOOS/KOOS scores as well as change scores after 1 year) of patients who were working preoperatively and had returned to work (*n* = 64 and *n* = 56) as compared to those who had not returned to work after 1 year and were not retired (*n* = 5 and *n* = 6), did not show any statistically significant differences for the THA and TKA patient groups, respectively.

## Discussion

This prospective study in patients undergoing THA and TKA showed that the large majority of patients who were working preoperatively returned to work 1 year after surgery. The mean time to return to work was 12 weeks. About 15–20 % of the patients returning to work worked less hours as compared to their preoperative work status. Only few patients under 65 years who were not working preoperatively were gainfully employed after 1 year.

Regarding the rate of working THA and TKA patients returning to work postoperatively, a comparison with the literature is hampered by the limited number of available studies, as well as by differences in study designs, in particular with respect to the selection of patients and duration of follow-up. A systematic review of the literature performed by our own group [6] showed that in the studies describing return to work, the proportions of patients returning to work ranged from 25 to 95 % at 1–12 months after THA (*n* = 7 studies) and from 71 to 83 % at 3–6 months after TKA (*n* = 2 studies) [6]. Only two studies included in this systematic review measured the proportion of patients returning to work at 1 year after surgery, both focused on THA patients. They showed that at 1 year after THA surgery 95/139 patients (68.3 %) [28] and 38/44 patients (86 %) [29] had returned to work, respectively. In addition, Sankar et al. [13] found that 87 % of working THA and 85 % of TKA patients had returned to work after 1 year. These results, from Bohm et al. [29] and Sankar et al. [13], are strikingly consistent with our results after 1 year (88 % in THA and 86 % in TKA). After the review was published, a retrospective study by Kievit et al. [14] showed that after a mean follow-up of 3.8 (1.3 SD) years after surgery 68 % of TKA patients had returned to work. It remains to be established to what extent this relatively lower proportion as compared to the present study was caused by patients not returning to work because of knee complaints or other reasons, such as the reaching the pensionable age. The most recent study, by Lombardi et al. [15], found a higher rate of 98 % of patients who underwent TKA returning to work. Even if those data were only compared with the TKA patients in our study, comparisons are seriously hampered by the observation that Lombardi et al. selected patients between 18 and 60 years of age and excluded patients with extensive medical comorbidities that would limit their activity level.

In the aforementioned systematic review [6] and a recent study by Lombardi et al. [15], the time of return to work after THA and TKA ranged from 1.1 to 10.5 weeks after THA (five studies) and 8.0–12.0 weeks after TKA (five studies) [6]. In comparison with these time periods, the mean time to return to work of 12 weeks as observed in the present study appears to be relatively long for THA. As the studies done so far were executed in different countries, it cannot be ruled out that the time of return to work may be dependent on the healthcare system as well as the social security system. In the Netherlands, sick leave from work is fully paid for during the first 2 years. Less favorable clinical outcomes are probably not likely to have played a role in the present study, as improvements of all clinical outcomes were in the same range as in other studies in unselected patients undergoing THA or TKA [20, 30–32]. To get more insight into the course of return to work in individual patients, more prospective studies measuring work status at multiple time points during the first year after surgery are needed.

Concerning the characteristics of patients who did and who did not return to work, no statistically significant differences were seen in the present study. On the one side, this could be related to the relatively small proportion of patients who did not return to work but is on the other hand consistent with the literature. A systematic review of the literature on determinants of return to work after THA and TKA found that only the surgical technique and the provision of movement restrictions to patients after surgery were related to return to work after THA [16].

An interesting finding of the present study which was, to our knowledge, not addressed in the literature was that postoperatively a considerable proportion of the THA and TKA patients worked less hours than before surgery. This loss of productivity does not seem to be counterbalanced by the relatively small numbers of patients who worked more hours than preoperatively and the numbers of patients who did not work preoperatively but were gainfully employed after 1 year. Our study showed some differences between working THA patients who did and who did not attain the number of hours they worked preoperatively. The number of hours working preoperatively was one of the factors, which is probably related to the a priori higher chance of losing working hours in patients who work more hours. The higher mean amount of working hours was in part due to some patients filling in more than the common maximum number of working hours in the Netherlands (36–40 h per week), indicating that this group may form a specific subgroup of patients. Larger patient groups are needed to confirm the findings of the present study and study the role of other factors that may have an impact on return to work, such as the characteristics of the surgery and rehabilitation, job characteristics including replacement of the patient’s position or tasks during his or her absence for the operation, or patient factors, such as a choice of the patient to stop working or decrease working hours (age close to retirement so patient decided to retire or work fewer hours). Given the growing number of relatively young and working patients undergoing THA or TKA, the absolute loss of work productivity on the national and international level could be considerable and warrants additional research involving multiple prospective cohorts in different countries on the reason for this loss of productivity at 1 year after THA and TKA surgery.

Our study showed that the characteristics of the total groups of patients undergoing THA and TKA were somewhat different, in particular with respect to BMI and educational level. It remains to be established to what extent the larger proportion of patients with a lower educational level in the TKA group (75/120; 63 %) as compared to the THA group (58/122; 48 %) is related to the physical demands of the job, in particular the knee demands. For that purpose, a study including an extensive assessment of the job characteristics and demands would be needed.

Our study has a number of limitations. The postoperative questionnaires were in a considerable proportion of patients returned incompletely, so that part of the data on postoperative work status needed to be gathered by means of a telephone interview. Moreover, irrespective of whether the data were obtained by questionnaire or telephone interview, the information was gathered partly retrospectively and is therefore prone to recall bias. Studies on return to work should preferably have a prospective design. We also employed 1 year as observation period, which is relatively long as compared to the average period of 12 weeks until return to work. In future research, applying more points for observation during the investigation period is advocated. In such research, information on postoperative complications such as infections, dislocations or deep venous thrombosis should also be recorded, as such events may have a large impact on the time until patients are able to return to their previous job.

In addition, the study concerned only patients undergoing surgery in one hospital in the Netherlands, whereas a multicenter study would have been preferable. Given the baseline characteristics of the patients including their radiographic characteristics as well as the magnitude of their clinical improvements over time, they appear, however, to be a fairly representative group of all patients with OA undergoing THA or TKA.

The strengths of our study are that we included patients with TKA, where research on work status in this patient group is scanty. Moreover, we gathered information on the number of working hours, showing a loss of work productivity despite high return to work rates.

In conclusion, this study shows that the large majority of working patients undergoing THA or TKA returns to work, after approximately 12 weeks. The present study suggests that apart from the small group of patients not returning to work, there may also be a group of patients who do return to work, yet not completely. Therefore, on the societal level, the total loss of productivity could be substantial given the large absolute numbers of patients undergoing total joint arthroplasties and warrants further analysis and intervention.
